# Listening in on Multicellular Communication in Human Tissue Immunology

**DOI:** 10.3389/fimmu.2022.884185

**Published:** 2022-05-13

**Authors:** Julian J. Albers, Karin Pelka

**Affiliations:** ^1^ Broad Institute of Massachusetts Institute of Technology (MIT) and Harvard, Cambridge, MA, United States; ^2^ Department of Medicine III, Klinikum rechts der Isar, Technical University of Munich, Munich, Germany; ^3^ Gladstone-University of California San Francisco (UCSF) Institute of Genomic Immunology, Gladstone Institutes, San Francisco, CA, United States

**Keywords:** human immunology, tissue biology, systems immunology, single cell, multicellularinteraction networks, cell-cell communication

## Abstract

Immune responses in human tissues rely on the concerted action of different cell types. Inter-cellular communication shapes both the function of the multicellular interaction networks and the fate of the individual cells that comprise them. With the advent of new methods to profile and experimentally perturb primary human tissues, we are now in a position to systematically identify and mechanistically dissect these cell-cell interactions and their modulators. Here, we introduce the concept of multicellular hubs, functional modules of immune responses in tissues. We outline a roadmap to discover multicellular hubs in human tissues and discuss how emerging technologies may further accelerate progress in this field.

## Introduction

The cells of our body do not function in isolation. Instead, they work together in spatially organized teams to ensure proper tissue function. Each cell relies on input from its surrounding microenvironment to fulfill its specific role and to contribute to the concerted function of its multicellular unit. For example, the proliferation and differentiation of stem cells is tightly regulated by their niche, as seen with Lgr5+ stem cells at the bottom of the intestinal crypt (1). Such spatial restriction of interactions is essential to keep tissue organization intact. Spatial organization is also important in the immune system, which consists of both tissue-resident cells and cells that circulate through the body and can dynamically come together in a coordinated manner. At sites of immune education, defined regions of the tissue enable specific cellular interactions through which lymphocytes acquire their functional capabilities, such as in the thymus where T cells mature (2), or lymph nodes where T and B cells get activated (3). In diseased tissues, immune cells assemble on demand to respond to threats and reconstitute homeostasis (4). Neutrophils swarm into acutely inflamed tissues by attracting each other to sites of injury, but also cross-inhibit their migration to prevent uncontrolled aggregation (5, 6). Tertiary lymphoid structures are examples of spatially restricted sites of immune cell interaction that can arise during chronic inflammation and cancer (7).

While some of the interactions governing tissue homeostasis, fate decisions, and immune cell education are well understood, we are just beginning to unveil the rules behind the cooperation between immune and non-immune cells in the diverse spectrum of human diseases. To understand how healthy tissues function, how diseased tissues are altered, and which perturbations could be exploited therapeutically, we need a simplified concept of how cells are organized in tissues. Furthermore, we need experimental and computational methods that systematically identify multicellular interaction networks and their components and mechanistically dissect their concerted multicellular behaviors.

Here, we describe the concept of multicellular hubs, which we define as dynamic, spatially proximal cells that interact and cooperatively enable specific tissue functions. We postulate that multicellular hubs represent functional modules of immune responses and that the functional state of healthy and diseased tissues can be described as a combination of those modules with varying activity, state, and localization. Finally, we outline a roadmap to systematically discover, experimentally model, and mechanistically dissect these modules.

## Multicellular Hubs - Functional Units of Immune Responses

Several recent publications support the idea that immune and tissue-resident non-immune cells are spatially organized into multicellular interaction networks in healthy and diseased tissues (8–10) including in tumors (11–16). The exact networks that were identified differed between studies, potentially due to differences in technologies and patient cohorts, and further investigation is required to understand if and how the interaction networks from different studies correspond to each other. However, profiling efforts of tumor biopsies suggest that a limited number of dominant multicellular interaction networks can explain even the large heterogeneity and variability seen in cancer (11, 14, 17, 18). Importantly, some of these multicellular interaction networks were furthermore found to be associated with survival (9, 12, 14–18).

**Figure 1:**
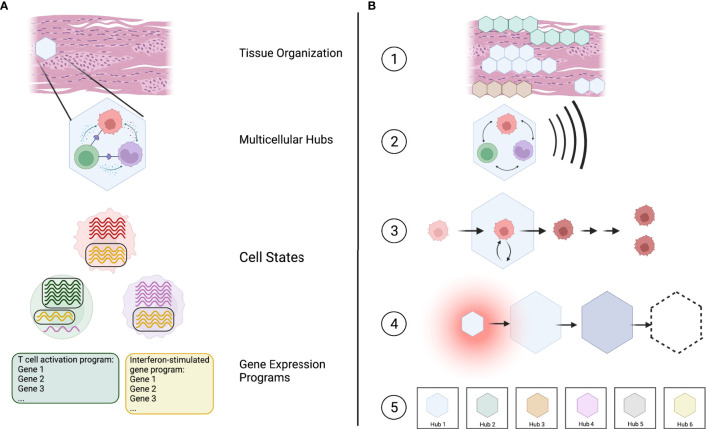
Multicellular hubs as coordinated modules in a hierarchical model of tissue organization and function. **(A)** Tissues can be viewed as a combination of multicellular hubs. Multicellular hubs are composed of interacting cells that express particular gene expression programs. **(B)** (1) The combination of type, activity, and location of different spatially organized hubs determines the functional state of the tissue. (2) The function of each hub arises from the concerted action of its cellular components. (3) The cells of the hub are influenced by their interaction with other cells in the hub. (4) Multicellular hubs are not static, but can change over time. (5) The number of different types of hubs is limited, with each type of hub potentially existing in different activation states analogous to cell types and their activation states.

Based on this, we suggest that multicellular hubs represent a valuable lens through which we can investigate immune responses in tissues. Multicellular hubs form an intermediate level in a hierarchical model of tissue organization that connects the molecular processes governing cellular behavior with the structure and functional state of the entire tissue. Specifically, we can now start to understand which genes function together in gene expression programs (19), how the combination of active gene expression programs defines a cell’s transcriptional state (20), which specific cell states interact to form multicellular hubs, and how the localization and combination of hubs defines the functional organization of a tissue (**Figure 1A**).

We postulate five key features of multicellular hubs (**Figure 1B**).

(1) Multicellular hubs are spatially organized units with a specific, limited function. The functional state of a tissue is defined by its composition of different multicellular hubs, their activity, and their location.
(2) The function of a multicellular hub arises from cellular cooperation, whereby each cell depends on the other cells to fulfill its specific task and contribute to the function of the hub.
(3) The interactions within multicellular hubs impact the cells of the hub by altering their cell-state trajectory and, in some cases, the developmental trajectory of their daughter cells.
(4) Multicellular hubs are not static. They form when specific environmental cues, such as chemokines that attract cells towards the same location, are present. Their cellular composition, size, and function can change over time, and they may eventually dissolve. For example, hubs may arise during physiological or pathological responses, change over the course of a disease, and disappear after treatment.
(5) The absolute number of different types ofmulticellular hubs is finite with each type of hub potentially existing in different activation states across healthy and diseased human tissues, analogous to cell types and their activation states. Given a sufficient sample size and the necessary methods, we can potentially catalog at least the different types of hubs in their entirety.

## How can we Identify Multicellular Hubs?

The advent of novel experimental technologies and computational methods provides us with an unprecedented opportunity to comprehensively map multicellular interaction networks in primary human tissues. Traditional hypothesis-driven approaches have been invaluable in building our current understanding of immune responses and tissue biology. However, they also have constraints that limit what we can learn from them. Firstly, these methods are usually limited to probing the interaction only between a specific subset of cell types of interest and cannot assemble a complete picture of the cellular interactions within tissues. Secondly, they often rely on the use of model organisms which do not always recapitulate human biology (21). Here, we describe how recent technological advances are now allowing us to systematically identify multicellular interaction networks in human tissues.

### Profiling of Primary Human Tissues

**Figure 2:**
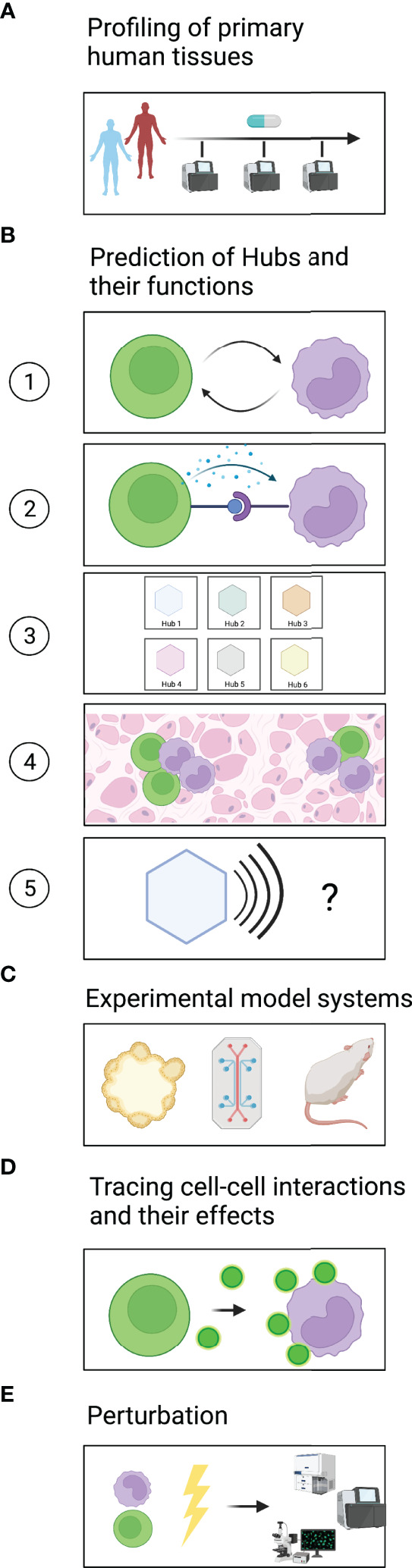
A roadmap to identify and mechanistically dissect multicellular hubs. **(A)** Profiling of healthy and diseased tissues across different timepoints using high-plex methods to discover multicellular hubs in primary human tissues. **(B)** Based on the profiling data, prediction of (1) which cells interact and (2) which signals they use. (3) Grouping of cell states into strongly connected modules to generate a dictionary of multicellular hubs. (4) Spatial map of hubs and their components in tissues. (5) Prediction of the hubs’ modular function. **(C)** Modeling of multicellular hubs in primary human ex vivo systems or animal models. **(D)** Experimental tracing of cell-cell communication. **(E)** Experimental perturbation to gain mechanistic understanding.

A major challenge in generating a comprehensive dictionary of multicellular hubs is access to clinically annotated primary human tissues (**Figure 2A**). The comparison of healthy and diseased tissues is vital to identify both steady-state and disease-specific hubs. Furthermore, a large number of samples will be required to identify rare multicellular hubs and assess their variability across patients. Ideally, tissues should be sampled from the same patients at different time points to evaluate how multicellular hubs change during the course of the disease or after treatment. For some diseased tissues, such as certain types of cancer or inflammatory bowel diseases, biopsies or resections are part of the clinical workup and are thus accessible to research. For the vast majority of human diseases longitudinal sampling of tissues remains impossible. However, insights from accessible sites and diseases might provide for example blood biomarkers that reflect how hubs develop in inaccessible tissues of interest.

To date, cellular interaction networks have been predicted based on high-plex profiling approaches (e.g., by single-cell RNA sequencing or CODEX) applied to relatively limited cohort sizes or by targeted low-plex profiling of large cohorts (e.g., by flow cytometry or immunohistochemistry) (8, 9, 11, 12, 15–17, 22–26). A growing number of studies are incorporating high-plex spatial information (27) to map cells and cellular interactions to specific locations within tissues. In the future, technological advances should make profiling methods more cost-efficient and scalable to large cohorts of human tissues, cover the full spectrum of analytes of a given modality (e.g., full transcriptome or full proteome), allow for single-cell resolution, and capture the spatial organization of the tissue.

To achieve a comprehensive picture, studies should furthermore integrate multiple different profiling modalities. Methods such as single-cell and single-nuclei RNA sequencing as well as single-cell ATAC sequencing are well established, while methods like single-cell proteomics are emerging to capture post-transcriptional changes in cell state (28). In addition, measuring non-cell autonomous characteristics of a tissue (e.g., microbiome, extracellular matrix) may be essential to fully understand tissue function. Pairing such relatively unbiased approaches with traditional methods like flow cytometry for established cell type markers or H&E staining to assess tissue histology will furthermore enable us to link newly gained global insights with the rich body of immunology literature.

### Computational Prediction of Hubs and Their Function

In the following sections, we outline how computational analyses can find signs of cellular interactions, predict possible ligand-receptor pairs that mediate these interactions, and define hubs (**Figure 2B**).

In recent years, a plethora of methods has been developed to infer cell-cell interaction from transcriptomic data (29) (**Figure 2B1**). Cellular interactions are commonly predicted based on the expression levels of known receptor-ligand pairs (30). Such an approach can identify which cells have the molecular equipment to interact and provide hypotheses regarding the directionality of the potential interactions. However, it does not address which interactions actually happened in the tissue analyzed. It also misses scenarios in which low receptor expression is sufficient to elicit a response, or in which activation of a receptor triggers its downregulation. The expression of target genes downstream of the engaged receptor in receiver cells together with ligand expression in donor cells (31) provides support as to which cells have likely interacted and functionally impacted each other. However, our knowledge of how different human cell types respond to specific stimuli is not yet complete. Signatures obtained through the increasing number of single-cell studies on primary human tissues should vastly improve the power of such approaches. Furthermore, targeted but spatially resolved measurements of ligands, receptors, and downstream targets could confirm receptor/ligand-based predictions regarding cellular interactions and the formation of multicellular hubs.

Methods that test for the co-variation of coordinated groups of genes or cell states across patient samples (11, 14, 17, 32) or space (16, 33, 34) do not rely on prior knowledge. Co-variation might indicate either that a common factor induces both signatures or that one signature induces another, representing a trace of cellular interaction. Querying the co-varying gene programs for enriched gene sets, transcription factor motifs, and receptor-ligand pairs can provide clues regarding the underlying mechanism and directionality of the interaction (**Figure 2B2**).

Once cellular interactions have been identified, graph-based methods e.g., (35, 36) can be used to group cell states into strongly connected modules, the multicellular hubs (**Figure 2B3**). It would also be interesting to use analysis methods that allow cell states or gene expression programs to be part of several multicellular hubs, executing different functions depending on the combination of interaction partners in each hub. With spatially-resolved methods, one can furthermore address how these hubs are organized within the tissue (**Figure 2B4**) and whether they are associated with histologically distinct regions.

Importantly, a dictionary of hubs only becomes valuable once we identify the function of each hub (**Figure 2B5**). Prior studies on genes, gene expression programs and cell types that compose the hub can help with its functional annotation. The location and spatial organization of the hub in the tissue, e.g., localization to areas of tissue damage or hypoxia, can provide further context regarding potential drivers or effects of the hub. Since the interpretation of high-plex omics datasets spans many areas of biology, input from experts in the respective fields is immensely helpful. Altogether, these steps create the dictionary of spatially organized hubs in primary human tissues that can guide and prioritize subsequent mechanistic studies.

## How can we Gain a Mechanistic Understanding of the Communication Pathways in Hubs and Determine the Hub’s Function?

### Experimental Model Systems

Experimental model systems are vital to validate computationally derived predictions (**Figure 2C**). In recent years, several model systems based on primary human cells have been developed, which are more likely than non-primary or non-human systems to recapitulate the signaling pathways occurring in human tissues, an important consideration for drug discovery (21). Organoids derived from patient samples, for example, retain inter-patient variability and can be used in co-cultures to study cellular interactions and test treatment responses *ex vivo* (12, 37, 38). To better capture heterogeneous cellular compositions, such as the tumor-microenvironment, patient-derived organotypic tumor spheroids (PDOTS) grown in microfluidic devices (39) and patient-derived organoids (PDOs) grown at an air-liquid interface (ALI) have been developed (40). Such culture systems can be used to study how perturbations including therapeutic agents used in the clinic impact cell-cell interactions and reshape multicellular hubs. Furthermore, Organ-on-a-chip systems can provide necessary physico-chemical cues present in primary tissues that may be important for multicellular hub formation, stability, and/or function (41).

While much progress has been made in developing these human ex vivo model systems, several limitations remain. Some cell types, e.g., neutrophils (42), have an inherently short life-span or cannot be cultured long enough to study intermediate- or long-term interactions. Furthermore, crucial environmental factors required for primary cell states to persist (38) or for cellular interactions to occur may be missing from these systems. In these cases, options currently available are to work with freshly isolated cells, mimic certain cell states experimentally (e.g., by cytokine stimulation or addition of physico-chemical stimuli), or switch to carefully chosen animal models. Independent of the model system, transcriptional or proteomic profiling of the cells should confirm the accurate resemblance to the cell states found in primary tissues.

### Tracing Cell-Cell Interactions and Their Effects

Several technologies to trace cell-cell interactions have been developed recently (**Figure 2D**) (43). These technologies test for proximity or direct contact between cells as an indicator of cellular interaction. One important consideration in choosing an approach is whether the interactions of interest are contact-dependent or contact-independent, e.g., through cytokines or chemokines, or both. Imaging-based technologies can assess both cells that are in direct contact with each other and cells that are close enough to communicate via paracrine signaling molecules. To study dynamic cellular interactions, e.g., between immune and non-immune cells in settings of acute injury or infection, live imaging-based readouts are particularly suitable (44–46).

Cell interactions can also be identified by methods that rely on the transfer of markers or signals between sender and receiver cells. These methods are suitable to study cell-cell interactions in living model organisms. However, tracing interactions in this way often requires genetic modifications of the sender and/or receiver cells, which makes it difficult to apply these technologies to primaryhuman model systems. Transferred markers can be chemicals [e.g., FucoID (47), LIPSTIC (48)], proteins [e.g., mCherry-niche (49), G-baToN (50)], or virally transmitted barcodes [e.g., RABIDseq (51)]. Alternatively, receiver cells can be equipped with genetically engineered receptors [e.g., SynNotch receptors (52)] that trigger the expression of a marker upon engaging in cell-cell interactions of interest. FucoID, LIPSTIC, G-baToN, and SynNotch receptors identify contact-dependent interactions, while mCherry-niche marks cells that are in close proximity to each other.

Depending on the research question, it might be necessary to not only identify which cell types interacted with each other, but to precisely know which individual cells were talking to each other. This can be accomplished by using imaging-based methods or methods that rely on the transfer of barcodes (e.g., RABIDseq). Furthermore, transcriptional, proteomic, or functional readouts can be used to demonstrate the impact of the observed interaction on the cells involved.

### Perturbation

Experimental perturbations are necessary to determine the directionality, mediators, and regulators of cell-cell interactions, and to identify which cellular components are needed for the concerted function of the hub (**Figure 2E**). In many cases, predictions of possible communication pathways can guide the choice of targeted perturbations, e.g., targeting specific ligand-receptor pairs. In other cases, a more comprehensive hypothesis-independent perturbation screen may be desirable to clarify the hub’s molecular circuits.

Different kinds of perturbations can be used to probe multicellular hubs. Recombinant ligands or cell supernatants from putative donor cells can be added to receiver cells to study individual cell-cell interactions. In multi-component hub model systems, individual cellular players can be added or depleted. Furthermore, genetic perturbations can be employed to turn signaling pathways off (CRISPR-mediated gene knockouts or interference) or on (CRISPR-mediated activation), identify key regulators, and when applied in co-culture systems, identify mediators of interaction (53–56). Importantly, these technologies are now becoming applicable to various primary human cell types (57–59). Lastly, small-molecule screens in organoids (60), PDOTs (39), ALI-PDOs (40), or Organ-on-a-chip systems (41) can be used to identify candidates for targeted therapeutic interventions (60).

In addition to the mode of perturbation, experimental readouts need to be carefully chosen to dissect how intra- and inter-cellular signaling pathways influence cellular behavior, hub formation, and hub function. Possible readouts include the measurement of markers of activated signaling pathways (61), the transcriptional state of single cells (62), assays of complex cellular phenotypes (63) and functions (53), the previously outlined methods to trace cell-cell interactions, or assessment of histological tissue organization (64). Together, these approaches can experimentally dissect computationally predicted multicellular hubs and provide a mechanistic understanding of the underlying molecular communication pathways and the concerted function they enable.

## Discussion

We predict that the number of ways that cells can interact and form multicellular hubs is finite. Given this hypothesis, it should be possible to map the entire multicellular hub space in healthy and diseased human tissues. Indeed, large-scale profiling efforts from consortia such as the Human Cell Atlas (HCA) (65), Human Tumor Atlas Network (HTAN) (66), Human Biomolecular Atlas Program (HuBMAP) (67), LifeTime Initiative (68) and others, are already in the process of generating and annotating dictionaries of cell types and states, gene expression programs, and ultimately also multicellular interaction networks. Furthermore, the toolbox of experimental model systems and technologies to trace and perturb cell-cell interactions is rapidly growing. Thus, not only profiling efforts but also mechanistic follow-up studies should become increasingly feasible at larger scales and in human model systems.

Thinking of tissues as a combination of multicellular hubs is certainly a simplification. Nevertheless, organizing cells and gene programs into hubs can help us to learn the cell- and context-specific function of genes, and point us to key regulators of physiologic and pathologic processes in human tissues. This is crucial in order to extract clinically relevant and therapeutically actionable knowledge from these increasingly complex data sets and derive universal principles of cellular interaction and cooperation.

## Open Questions

- Is the simplified view of tissues as a combination of multicellular hubs sufficient to describe the relevant immunologic processes in healthy and diseased tissues?
- How are cell trajectories influenced by the multicellular interactions within hubs?
- Is there a limited set of multicellular hubs that reoccur across different patients and diseases?
- Are the same hubs identified with different technologies?
- Which multicellular hubs are predictive of disease outcome and therapy responsiveness?
- Can disease-specific multicellular hubs be targeted therapeutically?

## Data Availability Statement

The original contributions presented in the study are included in the article/supplementary material. Further inquiries can be directed to the corresponding author.

## Author Contributions

JA and KP conceptualized, wrote, and reviewed the manuscript. All authors approved the submitted version.

## Funding

We are also thankful for the Stand Up to Cancer (SU2C) Peggy Prescott Early Career Scientist Award PA- 6146, SU2C Phillip A. Sharp Award SU2C-AACR-PS-32, BroadIgnite, and NIH/NCI R00CA259511 (to KP), and funds from the TUM Medical Graduate Center and the Studienstiftung des deutschen Volkes (to JA).

## Conflict of Interest

The authors declare that the research was conducted in the absence of any commercial or financial relationships that could be construed as a potential conflict of interest.

## Publisher’s Note

All claims expressed in this article are solely those of the authors and do not necessarily represent those of their affiliated organizations, or those of the publisher, the editors and the reviewers. Any product that may be evaluated in this article, or claim that may be made by its manufacturer, is not guaranteed or endorsed by the publisher.

## References

[1] Santos AJM Lo Y-H Mah AT Kuo CJ. The Intestinal Stem Cell Niche: Homeostasis and Adaptations. Trends Cell Biol (2018) 28:1062–78. doi: 10.1016/j.tcb.2018.08.001 PMC6338454 30195922

[2] Kumar BV Connors TJ Farber DL. Human T Cell Development, Localization, and Function Throughout Life. Immunity (2018) 48:202–13. doi: 10.1016/j.immuni.2018.01.007 PMC5826622 29466753

[3] Grant SM Lou M Yao L Germain RN Radtke AJ. The Lymph Node at a Glance - How Spatial Organization Optimizes the Immune Response. J Cell Sci (2020) 133:241828. doi: 10.1242/jcs.241828 PMC7063836 32144196

[4] Medzhitov R. The Spectrum of Inflammatory Responses. Science (2021) 374:1070–5. doi: 10.1126/science.abi5200 34822279

[5] Kienle K Lämmermann T. Neutrophil Swarming: An Essential Process of the Neutrophil Tissue Response. Immunol Rev (2016) 273:76–93. doi: 10.1111/imr.12458 27558329

[6] Kienle K Glaser KM Eickhoff S Mihlan M Knöpper K Reátegui E. Neutrophils Self-Limit Swarming to Contain Bacterial Growth *In Vivo* . Science (2021) 372:eabe7729. doi: 10.1126/science.abe7729 PMC8926156 34140358

[7] Schumacher TN Thommen DS. Tertiary Lymphoid Structures in Cancer. Science (2022) 375:eabf9419. doi: 10.1126/science.abf9419 34990248

[8] Guilliams M Bonnardel J Haest B Vanderborght B Wagner C Remmerie A. Spatial Proteogenomics Reveals Distinct and Evolutionarily Conserved Hepatic Macrophage Niches. Cell (2022) 185:379–6.e38. doi: 10.1016/j.cell.2021.12.018 PMC8809252 35021063

[9] Lake BB Menon R Winfree S Hu Q Ferreira RM Kalhor K. An Atlas of Healthy and Injured Cell States and Niches in the Human Kidney. bioRxiv (2021) 2021.07.28.454201. doi: 10.1101/2021.07.28.454201 PMC10356613 37468583

[10] Rodda LB Lu E Bennett ML Sokol CL Wang X Luther SA. Single-Cell RNA Sequencing of Lymph Node Stromal Cells Reveals Niche-Associated Heterogeneity. Immunity (2018) 48:1014–28.e6. doi: 10.1016/j.immuni.2018.04.006 PMC5971117 29752062

[11] Pelka K Hofree M Chen JH Sarkizova S Pirl JD Jorgji V. Spatially Organized Multicellular Immune Hubs in Human Colorectal Cancer. Cell (2021) 184:4734–52.e20. doi: 10.1016/j.cell.2021.08.003 PMC8772395 34450029

[12] Grünwald BT Devisme A Andrieux G Vyas F Aliar K McCloskey CW. Spatially Confined Sub-Tumor Microenvironments in Pancreatic Cancer. Cell (2021) 184:5577–92.e18. doi: 10.1016/j.cell.2021.09.022 34644529

[13] Casanova-Acebes M Dalla E Leader AM LeBerichel J Nikolic J Morales BM. Tissue-Resident Macrophages Provide a Pro-Tumorigenic Niche to Early NSCLC Cells. Nature (2021) 595:578–84. doi: 10.1038/s41586-021-03651-8 PMC8923521 34135508

[14] Luca BA Steen CB Matusiak M Azizi A Varma S Zhu C. Atlas of Clinically Distinct Cell States and Ecosystems Across Human Solid Tumors. Cell (2021) 184:5482–96.e28. doi: 10.1016/j.cell.2021.09.014 PMC8526411 34597583

[15] Keren L Bosse M Marquez D Angoshtari R Jain S Varma S. A Structured Tumor-Immune Microenvironment in Triple Negative Breast Cancer Revealed by Multiplexed Ion Beam Imaging. Cell (2018) 174:1373–87.e19. doi: 10.1016/j.cell.2018.08.039 PMC6132072 30193111

[16] Schürch CM Bhate SS Barlow GL Phillips DJ Noti L Zlobec I. Coordinated Cellular Neighborhoods Orchestrate Antitumoral Immunity at the Colorectal Cancer Invasive Front. Cell (2020) 182:1341–59.e19. doi: 10.1016/j.cell.2020.07.005 PMC7479520 32763154

[17] Combes AJ Samad B Tsui J Chew NW Yan P Reeder GC. Discovering Dominant Tumor Immune Archetypes in a Pan-Cancer Census. Cell (2022) 185:184–203.e19. doi: 10.1016/j.cell.2021.12.004 34963056 PMC8862608

[18] Thorsson V Gibbs DL Brown SD Wolf D Bortone DS Ou Yang T-H. The Immune Landscape of Cancer. Immunity (2018) 48:812–30.e14. doi: 10.1016/j.immuni.2018.03.023 PMC5982584 29628290

[19] Pope SD Medzhitov R. Emerging Principles of Gene Expression Programs and Their Regulation. Mol Cell (2018) 71:389–97. doi: 10.1016/j.molcel.2018.07.017 30075140

[20] Kotliar D Veres A Nagy MA Tabrizi S Hodis E Melton DA. Identifying Gene Expression Programs of Cell-Type Identity and Cellular Activity With Single-Cell RNA-Seq. Elife (2019) 8:e43803. doi: 10.7554/eLife.43803.044 31282856 PMC6639075

[21] Pulendran B Davis MM. The Science and Medicine of Human Immunology. Science (2020) 369:eaay4014. doi: 10.1126/science.aay4014 32973003 PMC7872131

[22] Smillie CS Biton M Ordovas-Montanes J Sullivan KM Burgin G Graham DB. Intra- and Inter-Cellular Rewiring of the Human Colon During Ulcerative Colitis. Cell (2019) 178:714–30.e22. doi: 10.1016/j.cell.2019.06.029 PMC6662628 31348891

[23] Zhang L Li Z Skrzypczynska KM Fang Q Zhang W O’Brien SA. Single-Cell Analyses Inform Mechanisms of Myeloid-Targeted Therapies in Colon Cancer. Cell (2020) 181:442–59.e29. doi: 10.1016/j.cell.2020.03.048 32302573

[24] Giraldo NA Berry S Becht E Ates D Schenk KM Engle EL. Spatial UMAP and Image Cytometry for Topographic Immuno-Oncology Biomarker Discovery. Cancer Immunol Res (2021) 9:1262–9. doi: 10.1158/2326-6066.CIR-21-0015 PMC8610079 34433588

[25] Gavrielatou N Liu Y Vathiotis I Zugazagoitia J Aung TN Shafi S. Association of PD-1/PD-L1 Co-Location With Immunotherapy Outcomes in Non-Small Cell Lung Cancer. Clin Cancer Res (2022) 28:360–7. doi: 10.1158/1078-0432.CCR-21-2649 PMC8776595 34686497

[26] Bindea G Mlecnik B Tosolini M Kirilovsky A Waldner M Obenauf AC. Spatiotemporal Dynamics of Intratumoral Immune Cells Reveal the Immune Landscape in Human Cancer. Immunity (2013) 39:782–95. doi: 10.1016/j.immuni.2013.10.003 24138885

[27] Lewis SM Asselin-Labat M-L Nguyen Q Berthelet J Tan X Wimmer VC. Spatial Omics and Multiplexed Imaging to Explore Cancer Biology. Nat Methods (2021) 18:997–1012. doi: 10.1038/s41592-021-01203-6 34341583

[28] Perkel JM. Single-Cell Proteomics Takes Centre Stage. Nature (2021) 597:580–2. doi: 10.1038/d41586-021-02530-6 34545225

[29] Armingol E Officer A Harismendy O Lewis NE. Deciphering Cell-Cell Interactions and Communication From Gene Expression. Nat Rev Genet (2021) 22:71–88. doi: 10.1038/s41576-020-00292-x 33168968 PMC7649713

[30] Efremova M Vento-Tormo M Teichmann SA Vento-Tormo R. CellPhoneDB: Inferring Cell–Cell Communication From Combined Expression of Multi-Subunit Ligand–Receptor Complexes. Nat Protoc (2020) 15:1484–506. doi: 10.1038/s41596-020-0292-x 32103204

[31] Browaeys R Saelens W Saeys Y. NicheNet: Modeling Intercellular Communication by Linking Ligands to Target Genes. Nat Methods (2020) 17:159–62. doi: 10.1038/s41592-019-0667-5 31819264

[32] Jerby-Arnon L Regev A. Mapping Multicellular Programs From Single-Cell Profiles. bioRxiv (2020):2020.08.11.245472. doi: 10.1101/2020.08.11.245472

[33] Fischer DS Schaar AC Theis FJ. Learning Cell Communication From Spatial Graphs of Cells. bioRxiv (2021) 2021.07.11.451750. doi: 10.1101/2021.07.11.451750

[34] Arnol D Schapiro D Bodenmiller B Saez-Rodriguez J Stegle O. Modeling Cell-Cell Interactions From Spatial Molecular Data With Spatial Variance Component Analysis. Cell Rep (2019) 29:202–11.e6. doi: 10.1016/j.celrep.2019.08.077 PMC6899515 31577949

[35] Blondel VD Guillaume J-L Lambiotte R Lefebvre E. Fast Unfolding of Communities in Large Networks. J Stat Mech (2008) 2008:P10008. doi: 10.1088/1742-5468/2008/10/P10008

[36] Esmailian P Jalili M. Community Detection in Signed Networks: The Role of Negative Ties in Different Scales. Sci Rep (2015) 5:1–17. doi: 10.1038/srep14339 PMC4585820 26395815

[37] Drost J Clevers H. Organoids in Cancer Research. Nat Rev Cancer (2018) 18:407–18. doi: 10.1038/s41568-018-0007-6 29692415

[38] Raghavan S Winter PS Navia AW Williams HL DenAdel A Lowder KE. Microenvironment Drives Cell State, Plasticity, and Drug Response in Pancreatic Cancer. Cell (2021) 184:6119–37.e26. doi: 10.1016/j.cell.2021.11.017 PMC8822455 34890551

[39] Jenkins RW Aref AR Lizotte PH Ivanova E Stinson S Zhou CW. Ex Vivo Profiling of PD-1 Blockade Using Organotypic Tumor Spheroids. Cancer Discovery (2018) 8:196–215. doi: 10.1158/2159-8290.CD-17-0833 29101162 PMC5809290

[40] Neal JT Li X Zhu J Giangarra V Grzeskowiak CL Ju J. Organoid Modeling of the Tumor Immune Microenvironment. Cell (2018) 175:1972–1988.e16. doi: 10.1016/j.cell.2018.11.021 30550791 PMC6656687

[41] Vunjak-Novakovic G Ronaldson-Bouchard K Radisic M. Organs-On-a-Chip Models for Biological Research. Cell (2021) 184:4597–611. doi: 10.1016/j.cell.2021.08.005 PMC8417425 34478657

[42] Blanter M Gouwy M Struyf S. Studying Neutrophil Function *In Vitro*: Cell Models and Environmental Factors. J Inflammation Res (2021) 14:141–62. doi: 10.2147/JIR.S284941 PMC7829132 33505167

[43] Bechtel TJ Reyes-Robles T Fadeyi OO Oslund RC. Strategies for Monitoring Cell-Cell Interactions. Nat Chem Biol (2021) 17:641–52. doi: 10.1038/s41589-021-00790-x 34035514

[44] McCarthy CE White JM Viola NT Gibson HM. *In Vivo* Imaging Technologies to Monitor the Immune System. Front Immunol (2020) 11:1067. doi: 10.3389/fimmu.2020.01067 32582173 PMC7280489

[45] Pittet MJ Garris CS Arlauckas SP Weissleder R. Recording the Wild Lives of Immune Cells. Sci Immunol (2018) 3:eaaq0491. doi: 10.1126/sciimmunol.aaq0491 30194240 PMC6771424

[46] Ronteix G Jain S Angely C Cazaux M Khazen R Bousso P. A Multiscale Immuno-Oncology on-Chip System (MIOCS) Establishes That Collective T Cell Behaviors Govern Tumor Regression. bioRxiv (2021) 2021.03.23.435334. doi: 10.1101/2021.03.23.435334

[47] Liu Z Li JP Chen M Wu M Shi Y Li W. Detecting Tumor Antigen-Specific T Cells via Interaction-Dependent Fucosyl-Biotinylation. Cell (2020) 183:1117–133.e19. doi: 10.1016/j.cell.2020.09.048 PMC7669731 33096019

[48] Pasqual G Chudnovskiy A Tas JMJ Agudelo M Schweitzer LD Cui A. Monitoring T Cell-Dendritic Cell Interactions *In Vivo* by Intercellular Enzymatic Labelling. Nature (2018) 553:496–500. doi: 10.1038/nature25442 29342141 PMC5853129

[49] Ombrato L Nolan E Passaro D Kurelac I Bridgeman VL Waclawiczek A. Generation of Neighbor-Labeling Cells to Study Intercellular Interactions *In Vivo* . Nat Protoc (2021) 16:872–92. doi: 10.1038/s41596-020-00438-5 PMC7615221 33311715

[50] Tang R Murray CW Linde IL Kramer NJ Lyu Z Tsai MK. A Versatile System to Record Cell-Cell Interactions. Elife (2020) 9:e61080. doi: 10.7554/eLife.61080 33025906 PMC7682987

[51] Clark IC Gutiérrez-Vázquez C Wheeler MA Li Z Rothhammer V Linnerbauer M. Barcoded Viral Tracing of Single-Cell Interactions in Central Nervous System Inflammation. Science (2021) 372:eabf1230. doi: 10.1126/science.abf1230 33888612 PMC8157482

[52] Morsut L Roybal KT Xiong X Gordley RM Coyle SM Thomson M. Engineering Customized Cell Sensing and Response Behaviors Using Synthetic Notch Receptors. Cell (2016) 164:780–91. doi: 10.1016/j.cell.2016.01.012 PMC4752866 26830878

[53] Kamber RA Nishiga Y Morton B Banuelos AM Barkal AA Vences-Catalán F. Inter-Cellular CRISPR Screens Reveal Regulators of Cancer Cell Phagocytosis. Nature (2021) 597:549–54. doi: 10.1038/s41586-021-03879-4 PMC9419706 34497417

[54] Patel SJ Sanjana NE Kishton RJ Eidizadeh A Vodnala SK Cam M. Identification of Essential Genes for Cancer Immunotherapy. Nature (2017) 548:537–42. doi: 10.1038/nature23477 PMC5870757 28783722

[55] Manguso RT Pope HW Zimmer MD Brown FD Yates KB Miller BC. *In Vivo* CRISPR Screening Identifies Ptpn2 as a Cancer Immunotherapy Target. Nature (2017) 547:413–8. doi: 10.1038/nature23270 PMC5924693 28723893

[56] Kearney CJ Vervoort SJ Hogg SJ Ramsbottom KM Freeman AJ Lalaoui N. Tumor Immune Evasion Arises Through Loss of TNF Sensitivity. Sci Immunol (2018) 3:eaar3451. doi: 10.1126/sciimmunol.aar3451 29776993

[57] Shifrut E Carnevale J Tobin V Roth TL Woo JM Bui CT. Genome-Wide CRISPR Screens in Primary Human T Cells Reveal Key Regulators of Immune Function. Cell (2018) 175:1958–71.e15. doi: 10.1016/j.cell.2018.10.024 PMC6689405 30449619

[58] Hiatt J Cavero DA McGregor MJ Zheng W Budzik JM Roth TL. Efficient Generation of Isogenic Primary Human Myeloid Cells Using CRISPR-Cas9 Ribonucleoproteins. Cell Rep (2021) 35:109105. doi: 10.1016/j.celrep.2021.109105 33979618 PMC8188731

[59] Schmidt R Steinhart Z Layeghi M Freimer JW Bueno R Nguyen VQ. CRISPR Activation and Interference Screens Decode Stimulation Responses in Primary Human T Cells. Science (2022) 375:eabj4008. doi: 10.1126/science.abj4008 35113687 PMC9307090

[60] Driehuis E Kretzschmar K Clevers H. Establishment of Patient-Derived Cancer Organoids for Drug-Screening Applications. Nat Protoc (2020) 15:3380–409. doi: 10.1038/s41596-020-0379-4 32929210

[61] Schraivogel D Kuhn TM Rauscher B Rodríguez-Martínez M Paulsen M Owsley K. High-Speed Fluorescence Image-Enabled Cell Sorting. Science (2022) 375:315–20. doi: 10.1126/science.abj3013 PMC7613231 35050652

[62] Dixit A Parnas O Li B Chen J Fulco CP Jerby-Arnon L. Perturb-Seq: Dissecting Molecular Circuits With Scalable Single-Cell RNA Profiling of Pooled Genetic Screens. Cell (2016) 167:1853–66.e17. doi: 10.1016/j.cell.2016.11.038 PMC5181115 27984732

[63] Feldman D Singh A Schmid-Burgk JL Carlson RJ Mezger A Garrity AJ. Optical Pooled Screens in Human Cells. Cell (2019) 179:787–99.e17. doi: 10.1016/j.cell.2019.09.016 PMC6886477 31626775

[64] Dhainaut M Rose SA Akturk G Wroblewska A Park ES Nielsen SR. Perturb-Map Enables CRISPR Genomics With Spatial Resolution and Identifies Regulators of Tumor Immune Composition. bioRxiv (2021) 2021.07.13.451021. doi: 10.1101/2021.07.13.451021

[65] Regev A Teichmann SA Lander ES Amit I Benoist C Birney E. The Human Cell Atlas. Elife (2017) 6:e27041. doi: 10.7554/eLife.27041 29206104 PMC5762154

[66] Rozenblatt-Rosen O Regev A Oberdoerffer P Nawy T Hupalowska A Rood JE. The Human Tumor Atlas Network: Charting Tumor Transitions Across Space and Time at Single-Cell Resolution. Cell (2020) 181:236–49. doi: 10.1016/j.cell.2020.03.053 PMC7376497 32302568

[67] HuBMAP Consortium. The Human Body at Cellular Resolution: The NIH Human Biomolecular Atlas Program. Nature (2019) 574:187–92. doi: 10.1038/s41586-019-1629-x PMC6800388 31597973

[68] Rajewsky N Almouzni G Gorski SA Aerts S Amit I Bertero MG. LifeTime and Improving European Healthcare Through Cell-Based Interceptive Medicine. Nature (2020) 587:377–86. doi: 10.1038/s41586-020-2715-9 PMC7656507 32894860

